# Biosynthesis of novel cannabigerolic acid derivatives by engineering the substrate specificity of aromatic prenyltransferase

**DOI:** 10.3389/fbioe.2025.1563708

**Published:** 2025-04-10

**Authors:** Hoe-Suk Lee, Jisu Park, Taejung Kim, Huitae Min, Seongsu Na, Soon Young Park, Young-Tae Park, Young Joo Yeon, Jungyeob Ham

**Affiliations:** ^1^ Department of Biochemical Engineering, Gangneung-Wonju National University, Gangneung, Republic of Korea; ^2^ Natural Product Research Center, Korea Institute of Science and Technology (KIST), Gangneung, Republic of Korea; ^3^ Division of Natural Product Applied Science, University of Science and Technology (UST), Daejeon, Republic of Korea; ^4^ NeoCannBio Co., Ltd., Seoul, Republic of Korea

**Keywords:** cannabinoid, cannabigerolic acid derivative, aromatic prenyltransferase NphB, computational enzyme design, engineered *E. coli* whole cell, biocatalytic system

## Abstract

**Introduction:**

Cannabinoids possess significant therapeutic potential, but their natural chemical diversity derived from plant biosynthesis is limited. Efficient biotransformation processes are required to expand the range of accessible cannabinoids. This study aimed to enhance the selective biosynthesis of cannabigerolic acid (CBGA) and its derivatives with varying aliphatic chain lengths, which serve as key precursors to various cannabinoids.

**Methods:**

We employed computational modeling and structure-guided mutagenesis to engineer the aromatic prenyltransferase NphB. Mutants were designed via *in silico* docking analyses to optimize substrate orientation and catalytic distance. The variants were expressed in *E. coli*, and their catalytic efficiencies were evaluated through *in vivo* whole-cell and *in vitro* enzymatic assays. Products were identified and quantified by UHPLC-MS.

**Results:**

Engineered NphB variants exhibited significant improvements, with triple mutants achieving a 7-fold increase in CBGA production and a 4-fold increase in cannabigerovarinic acid production. Additionally, a single mutant also enhanced the synthesis of 3-geranyl orsellinic acid by 1.3-fold. Notably, novel enzymatic activity was identified that enabled the biosynthesis of 3-geranyl-2,4-dihydroxybenzoic acid. Structural analyses revealed that the mutations improved the spatial positioning of aromatic substrates relative to the co-substrate geranyl pyrophosphate.

**Discussion:**

This study demonstrates the feasibility of enzyme design to tailor prenyltransferase specificity for the production of diverse CBGA derivatives. These findings lay the groundwork for the microbial production of novel cannabinoids and offer promising potential for the development of scalable biocatalytic systems for therapeutic and industrial applications.

## 1 Introduction

*Cannabis sativa*, also known as marijuana, is an annual plant believed to have originated from China and Central Asia whose seeds and leaves have been utilized for centuries as sources of textile fiber, paper, cosmetics, fuels, and herbal medicine (Russo et al., 2008; Small, 2015; Andre et al., 2016). Historically, cannabis has been employed in the treatment of rheumatic pain, intestinal constipation, inflammation, menstrual disorders, and malaria (Hill et al., 2017). More recently, its therapeutic potential has been demonstrated for managing neuropathies such as epilepsy, schizophrenia, brain tumors, Parkinson’s disease, and multiple sclerosis (Di Marzo, 2008; More and Choi, 2015; Gray and Whalley, 2020; Mecha et al., 2020; Held-Feindt et al., 2006). However, the psychoactive side effects of cannabis, including disorientation, hallucination, impaired memory, nausea, and depression, have necessitated strict regulations on its use. The adverse effects of cannabis are well documented (Wang et al., 2008). To harness the therapeutic potential of cannabinoids while mitigating their psychoactive and adverse effects, it is critical to isolate beneficial cannabinoids from those with undesirable properties. This process is complicated by the structural and physicochemical similarities among cannabinoids (Hazekamp et al., 2004). Furthermore, the isolation of rare cannabinoids present in low abundance poses additional challenges, underscoring the need for biotransformation strategies capable of efficiently producing specialized cannabinoids.

Over 120 phytocannabinoids sharing a common C21 terpenophenolic backbone have been identified and categorized into 11 sub-classes, including cannabigerol (CBG), cannabidiol (CBD), cannabichromene (CBC), cannabielsoin (CBE), and (−)-Δ^9^-tetrahydrocannabinol (Δ^9^-THC) (Piscitelli and Di Marzo, 2021; Radwan et al., 2021; Lim et al., 2022). Cannabigerolic acid (CBGA) is a central precursor in the cannabinoid metabolic pathway, serving as the substrate for enzymatic conversion into cannabidiolic acid (CBDA), cannabichromenic acid (CBCA), and (−)-Δ^9^-tetrahydrocannabinolic acid (THCA) through specific synthase enzymes (Morimoto et al., 1998; Lange et al., 2016; Dai et al., 2024). Subsequent decarboxylation produces the corresponding cannabinoids CBD, CBC, and THC (Lange and Zager, 2022). Diversifying CBGA derivatives as potential substrates, therefore, directly expands the range of cannabinoids that can be recombinantly synthesized.

The binding affinity and biological activity of cannabinoids are influenced by the aliphatic chain length on the C6 atom of the olivetolic ring (Bow and Rimoldi, 2016). For example, cannabinoids with shorter aliphatic chains than CBD (with a five-carbon chain) exhibit a reduced ability to inhibit cAMP accumulation via the G protein-coupled receptor GPR12 (Brown et al., 2017). Derivatives such as cannabidivarin (CBDV) and cannabigerovarin (CBGV), which possess three-carbon chains, exhibit significant activity on transmembrane cation channels that are implicated in neuropathic pain, inflammation, and respiratory disorders (Muller et al., 2019).

**SCHEME 1:**
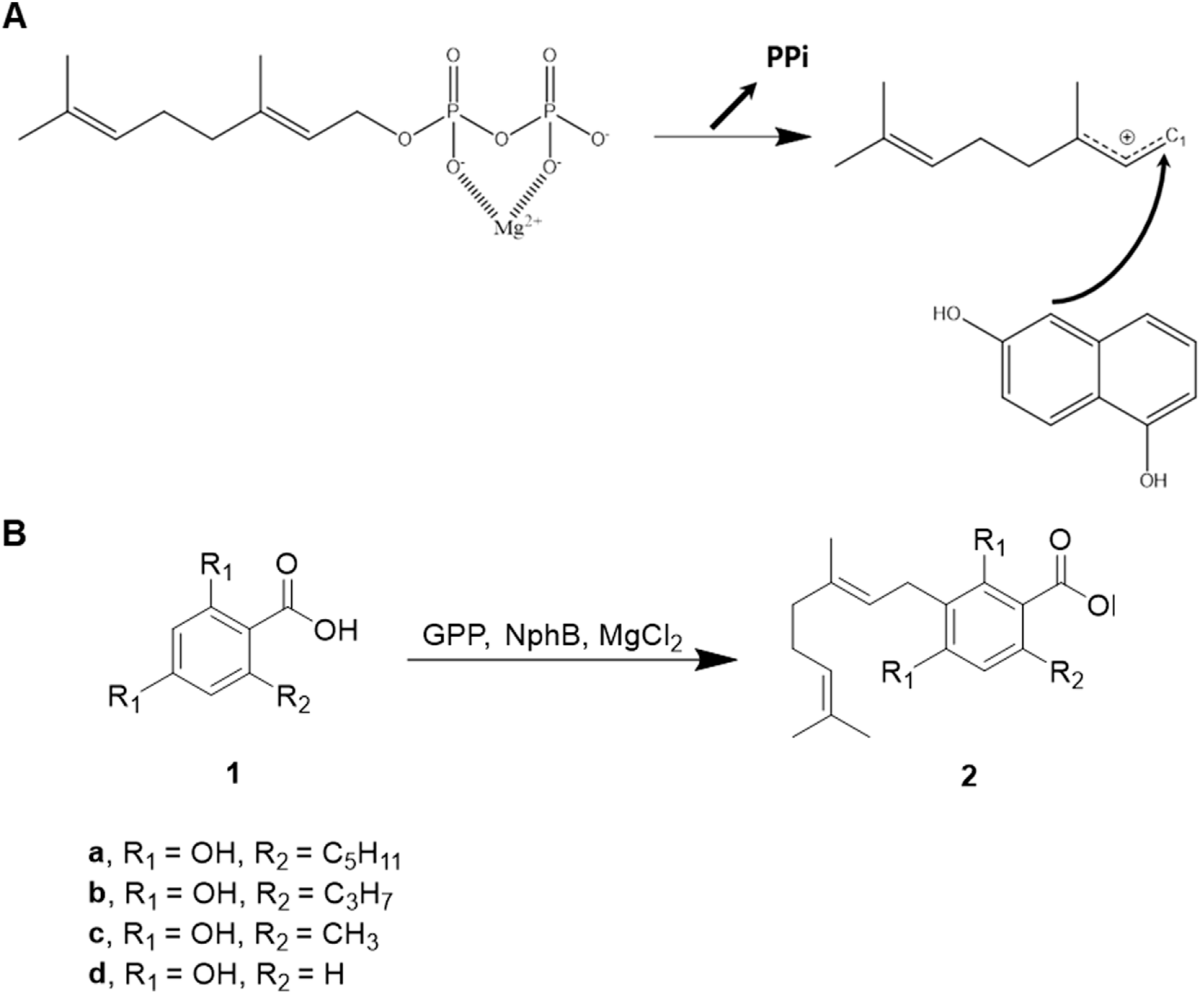
NphB-mediated biosynthesis of CBGA derivatives. **(A)** Reaction mechanism proposed from the crystal structure of NphB (PDB ID 1ZB6) co-crystalized with geranyl S-thiolodiphosphate and 1,6-dihydroxy naphthalene. **(B)** The aromatic substrates and the geranylated products differ by the length of the carbon chains at R_2_ (**1a**, olivetolic acid; **2a**, CBGA; **1b**, varinolic acid; **2b**, CBGVA; **1c**, orsellinic acid; **2c**, 3-geranyl orsellinic acid; **1d**, 2,4-dihydroxybenzoic acid**; 2d**, 3-geranyl 2,4-dihydroxybenzoic acid).

CBGA biosynthesis involves the transfer of the isoprenoid group from geranyl pyrophosphate (GPP) to aromatic olivetolic acid, catalyzed by aromatic prenyltransferase enzymes (Qian et al., 2019; Valliere et al., 2019; Lim et al., 2022; Spitzer et al., 2023). While most of these enzymes are membrane-bound plant proteins, the discovery of the soluble NphB enzyme from *Streptomyces* offers a promising alternative for recombinant microbial expression and engineering (Kuzuyama et al., 2005). The proposed reaction mechanism of NphB suggests a carbon-mediated nucleophilic attack on C1 of GPP, with the negative charge of the pyrophosphate moiety stabilized by Mg^2+^ in an SN2-like manner, followed by carbocation-mediated electrophilic capture (Scheme 1A). In the original crystal structure by Kuzuyama and coworkers (PDB ID: 1ZB6), the distance between C1 of geranyl S-thiolodiphosphate (GST), an analog of GPP, and the prenylation site on 1,6-dihydroxy naphthalene (1,6-DHN) was described as a key factor for the reaction mechanism. It was shown to be 4 Å. NphB exhibits broad substrate specificity for small aromatic compounds, making it an excellent engineering target for producing various CBGA derivatives (Yang et al., 2012).

In this study, we present computationally designed NphB mutants tailored for the biosynthesis of CBGA derivatives with variable aliphatic chain lengths on the C6 atom. These derivatives include CBGA (five-carbon chain), cannabigerovarinic acid (CBGVA, three-carbon chain), 3-geranyl orsellinic acid (one-carbon chain), and 3-geranyl 2,4-dihydroxybenzoic acid (zero-carbon chain) (Scheme 1B, **2a**–**2d**). The designed mutants were screened based on their CBGA derivative yields, enzymatic activities were confirmed *in vitro*, and reaction conditions were optimized to enhance production. This work establishes a foundation for expanding the cannabinoid repertoire through enzyme engineering, with potential applications in therapeutic and industrial contexts.

## 2 Materials and methods

### 2.1 Computational design and structural analysis of NphB variants

The *in silico* models of NphB wild-type (WT), NphB*, and other variants were generated from the crystal structure of NphB (PDB: 1ZB6) with Maestro software (Release 2022-1, Schrödinger, LLC, New York, NY). The structure was stripped of the 1,6-DHN and GST ligands, followed by a protein preparation process including assigning bond orders, adding hydrogens, creating zero-order bonds to Mg^2+^, filling in missing side chains and loops, generating het states using Epik, optimizing H-bonds using PROPKA, and restraining and prime minimizing using an OPLS3e forcefield to create energetically stable models. Receptor grids were created at the GST and 1,6-DHN coordinates for the docking of GPP and the aromatic substrate **1a**∼**1d**, respectively. Sufficient volumes were allowed for the receptor grids to encompass the active site. Ligands were prepared via the LigPrep module, generating possible ionization states at the target pH and tautomers with an OPLS3e forcefield. Ligand docking in the receptor grids was carried out using the standard precision mode of the Glide module with flexible ligand sampling. Options such as reward intramolecular hydrogen bonds and enhanced planarity of conjugated pi groups were applied, and all other settings were set as default. Visualization of the models was carried out using the Pymol Molecular Graphics System (Version 2.0, Schrödinger, LLC, New York, NY).

### 2.2 Cloning and site-directed mutagenesis

The NphB gene was synthesized from Cosmogenetech (Seoul, Korea) with codon optimization for *Escherichia coli*. The NphB gene was transformed into *E. coli* DH5α for cloning and mutagenesis (Supplementary Table S1). The NphB was cloned in the pET 22b (+) vector by In-Fusion^®^ HD Cloning Kit (Takara, Kusatsu, Japan) with an N-terminal His-tag. The NphB gene was mutated following the QuikChange II Site-Directed Mutagenesis protocol (Agilent, Santa Clara, United States).

### 2.3 Protein expression and purification

The plasmids were transformed into the expression host *E. coli* BL21 (DE3) strain and selected on an LB-agar plate supplemented with 100 μg mL^−1^ ampicillin at 37°C. Selected transformants were inoculated in 5 mL LB media with 100 μg mL^−1^ ampicillin at 37°C and 200 rpm under aerobic conditions overnight. For protein expression purposes, 5 mL of the preculture was used to inoculate 200 mL cell culture, which was grown to reach an optical density up to 0.5 at 600 nm (OD_600_) and induced with 0.8 mM isopropyl β-D-1-thiogalactopyranoside (IPTG) at 20°C, 150 rpm for 20 h. The induced cell cultures were harvested by centrifugation at 3,000 g, 4°C for 20 min. The harvested cells were either directly applied to whole-cell bioconversion for the biosynthesis of CBGA derivatives or lysed by Bugbuster^®^ (Merck Millipore, Billerica, United States), centrifuged (8,000 g, 4°C, 20 min) for cell debris removal, subjected to Ni-affinity chromatography (Qiagen, Hilden, Germany) for elution with elution buffer (50 mM Tris-HCl, 300 mM NaCl, 250 mM imidazole), and concentrated using Amicon Ultra 15 mL centrifugal filters with a 10 kDa pore size. Purification of the enzymes to homogeneity was confirmed by 12% SDS-PAGE. The concentrations of the purified enzymes were determined by the NanoDrop Protein Quantification.

### 2.4 *In vivo* whole-cell conversion and *in vitro* enzymatic conversion

For the whole-cell-mediated biosynthesis of CBGA derivatives, the harvested cells were washed with distilled water. The reactions were performed in 50-mL tubes with 2 mL reaction mixtures, each consisting of 50 mM Tris-HCl buffer (pH 8.0), 1 mM aromatic substrates **1a**∼**1d**, 1 mM GPP, 5 mM MgCl_2_, and 0.9 g_cdw_ L^−1^ of cells at 30°C, 200 rpm. After 24 h, 100 µL of the culture was sampled. The samples were resuspended in 900 µL of methanol, centrifuged for 5 min at 16,000 g, and filtered using a 0.2-μm polyvinylidene fluoride (PVDF) filter from SMARTiLab (Rabat, Morocco). The reaction mixture for the *in vitro* enzymatic conversion consisted of 5 µM purified NphB variants, 1 mM aromatic substrates, 1 mM GPP, 5 mM MgCl_2_, and 50 mM Tris-HCl buffer (pH 8.0) in a total volume of 2 mL at 30°C, 200 rpm. Aliquots (100 μL) of the mixture were sampled at 0 h, 1 h, 3 h, 6 h, and 9 h. The samples were resuspended in 900 µL of methanol, centrifuged for 5 min at 16,000 g, and filtered by the 0.2 μm PVDF filter.

### 2.5 Analytical methods

The filtrate was introduced into a Shimadzu Nexera X2 UHPLC system comprised of a solvent degassing unit (DGU–20A), binary pump (LC–30AD), autosampler (SIL–30AC), system controller unit (CBM–20A), photodiode array detector (SPD–M20A), and column oven unit (CTO–20AC) for qualitative and or quantitative analysis. Electrospray ionization (ESI)-mass spectrometry (MS) (Shimadzu LCMS-2020 system) was used for qualitative analysis. A Phenomenex Luna Omega polar C18 column (150 mm × 2.1 mm, 1.6 μm) was used for the compound separation. The mobile phase contained a binary gradient of solvent A (water) and solvent B (MeCN), as follows: initially, 70%; 10 min, 85%; 11 min, 95%; and 15 min, 70% for solvent B. The flow rate was established to 0.3 mL min^−1^, and a detection wavelength of 220 nm was used.

## 3 Results

### 3.1 Modeling and rational design of NphB variants for the biosynthesis of the CBGA derivatives

NphB variants were previously developed to increase the enzyme specificity for the CBGA against 2-O-geranyl-olivetolic acid, which is a major side product generated from the nonspecific prenylation of the O2 position on olivetolic acid (**1a**), and an NphB G286S/Y288A double mutant was found to be the most potent for this purpose (Qian et al., 2019; Valliere et al., 2019). Therefore, it was designated as NphB* and chosen for further design to adapt to the synthesis of CBGA derivatives. The X-ray crystal structure of NphB (PDB ID: 1ZB6), bound to geranyl S-thioldiphosphate (GST), 1,6-dihydroxynaphthalene (1,6-DHN), and Mg^2^, was used as the reference structure. The ligands are an unreactive analog of geranyl diphosphate (GPP), an aromatic substrate, and a metal cofactor, respectively, positioned in the active site at the center of an α/β-barrel fold. The structure was stripped of GST and 1,6-DHN and mutated to Y288A/G286S to model the NphB* structure *in silico*. Previous structure-guided mutation designs on the NphB used the native GST in the crystal structure for modeling, whereas GPP and the aromatic substrates **1a**∼**1d** were sequentially docked in this study (Valliere et al., 2019; Lim et al., 2022).

Docking of GPP led to a conformation similar to the GST of the crystal structure, with the Gibbs free energy of binding (ΔG_bind_) of −13.474 kcal mol^−1^ (Supplementary Figure S1) (Kuzuyama et al., 2005; Yang et al., 2012). The multiple negatively charged diphosphate moiety was stabilized by a network of salt bridges involving several positively charged residues such as K119, K169, R228, and K284, as well as Mg^2+^ cofactor, which was found to be necessary for NphB activity. This interaction was reinforced by hydrogen bonds involving the hydrophilic side chains of S51, Y121, N173, Y175, Y216, and T218. The geranyl moiety protrudes deeply into a hydrophobic pocket composed of V47, V49, A108, F123, and M162. Several hydrophilic residues, including S64, S66, and S286, were also observed, which may be subjected to engineering for enhanced binding of the GPP substrate.

**FIGURE 1:**
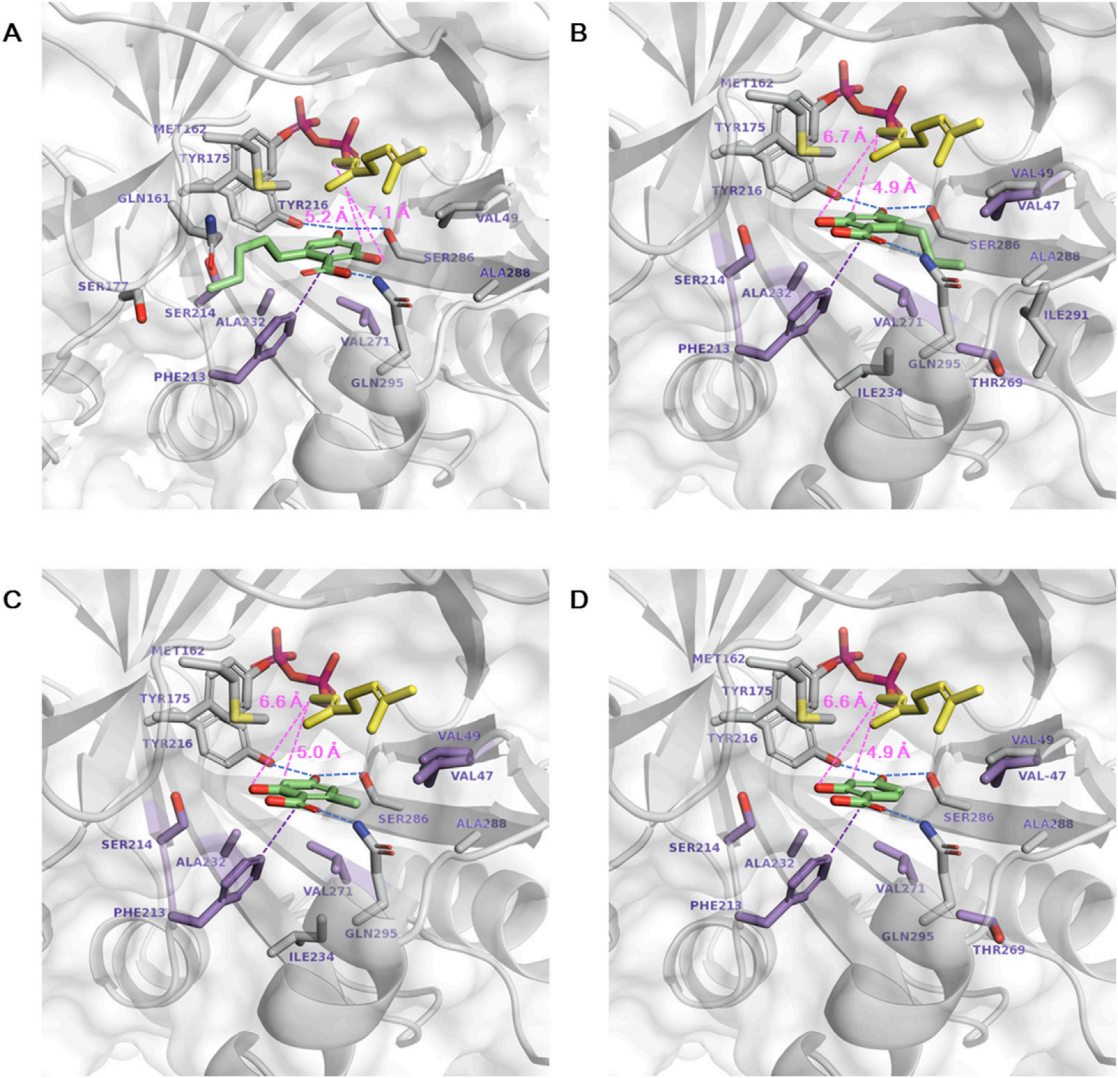
Docking of aromatic substrates in the NphB* active site. **(A–D)** correspond to the docking of **1a∼1d**, respectively. The stick representations are GPP (yellow), aromatic substrate (green), mutation target residues (purple), and other substrate-interacting residues (gray). Hydrogen bonds, π–π interactions, and the catalytic distances between the C1 atom of GPP and the C3 or O2 atoms of the aromatic substrates are marked in dotted blue, purple, and pink lines, respectively.

Subsequent docking of each aromatic substrate (**1a**∼**1d)** resulted in multiple ligand binding poses. The most productive binding pose was identified based on the lowest ΔG_bind_ and the minimal catalytic distance between the C1 of GPP and the C3 of substrate **1a**∼**1d** for prenylation (Figure 1). In the selected poses, the distances from the C1 of GPP to the C3 of **1a**∼**1d** were shorter than those to the O2 of **1a**∼**1d**, supporting the regioselectivity of C3 prenylation. Mutation targets were selected with the aim of enhancing the binding affinity of **1a**∼**1d** and reducing the catalytic distance by bringing the substrates closer to GPP. In the most stable and productive binding pose of **1a**, the catalytic distance was 5.2 Å. The aromatic ring formed a π–π interaction with F213, while the carboxylic acid was stabilized by Q295. Additionally, the O4 atom was stabilized by Y216 and S286 through hydrogen bonds (Figure 1A). The long aliphatic chain extended toward the entrance of the active site near M162 and S214. Primary mutation targets lie below the plane of the aromatic ring of **1a**, including A232I/L/M/V, F213Y, S214T, and V271F, which are expected to introduce larger residues and push **1a** toward GPP to facilitate a covalent bond formation.

The binding conformations of **1b**∼**1d** were similar to **1a** in that the π–π interaction between the aromatic ring and F213, the stabilization of the carboxylic acid by Q295, and the hydrogen bonds between the O4 atom, Y216, and S286 were observed (Figures 1B–D). These interactions may be the predominant factor in determining the substrate binding conformation regardless of the length of the aliphatic chain. The only difference between **1b**∼**1d** and **1a** was the direction of the aliphatic chain, which flipped to the opposite direction of the active site entrance toward A288, T269, and I291. This could be enabled by the mutation of Y288A, which excavated deep in the binding pocket to accommodate the chain of zero to three carbons, but the pocket volume was not sufficient for the five-carbon chain of **1a**. In addition to the mutation targets selected for **1a**, sites were selected in this direction to enhance the hydrophobic interaction with the aliphatic chains in **1b**∼**1d**, namely, T269I/L/V and V47A for **1b**, V47I/L/M and V49I/L/M for **1c**, and T269I/L/M and V47I/L/M for **1d**. Aliphatic amino acids of various sizes were employed to investigate the hydrophobic effect. V47K was also designed for **1d,** as the substrate does not have an aliphatic chain at the C6 position, and a direct cation- π interaction is available.

### 3.2 Screening of NphB* variants for the production of the CBGA derivatives

**FIGURE 2:**
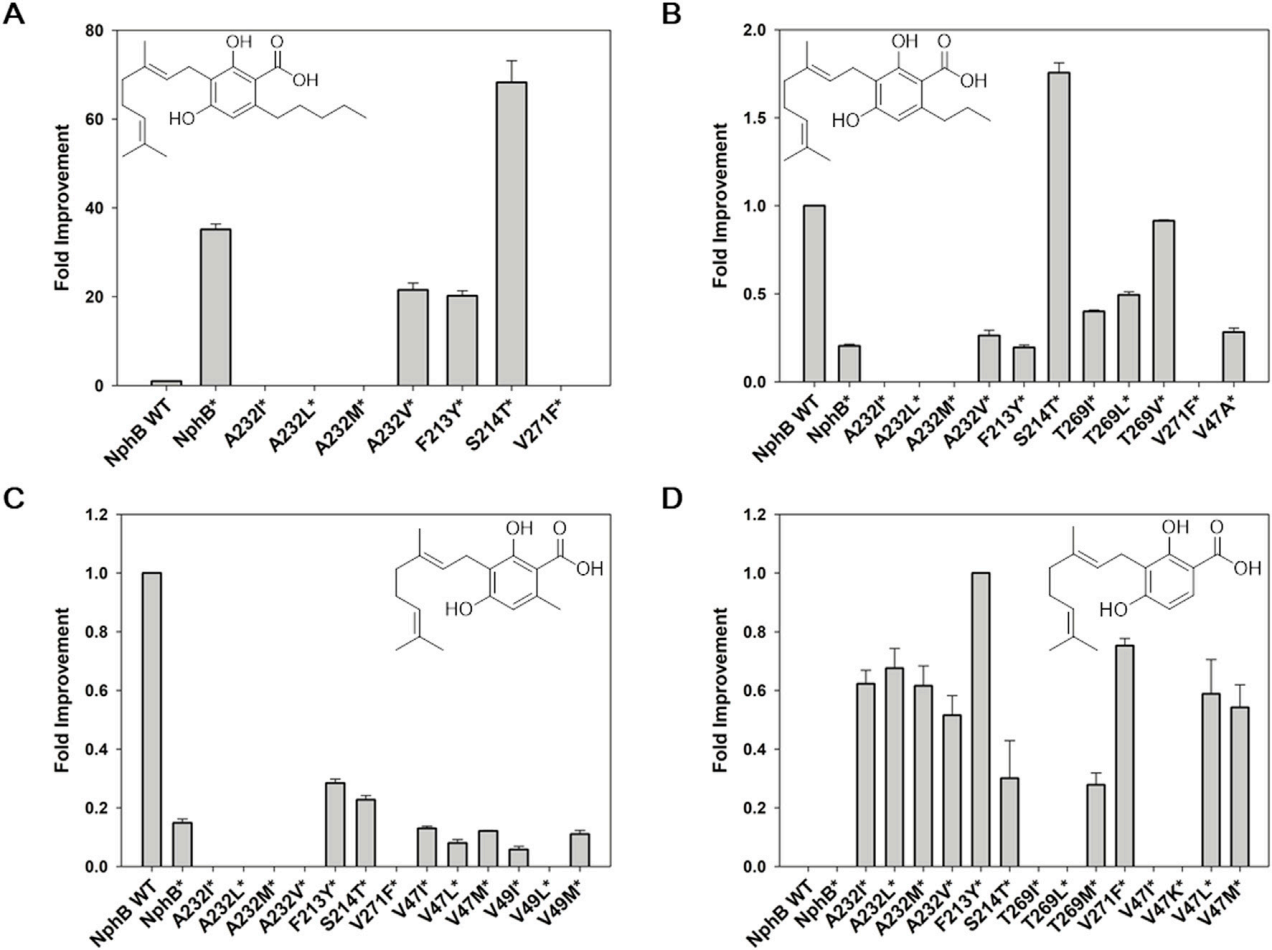
Screening NphB* variants for the biotransformation of the CBGA derivatives. **(A–C)**, fold improvement in the yields for **2a**∼**2c**, respectively, relative to the yield of NphB WT; **(D)** fold improvement in the yields for **2d**, relative to the yield of NphB* F223Y (no biotransformation activity was detected for **2d** in NphB WT or NphB*). The reaction was carried out at 30°C in a 50 mM Tris-HCl buffer (pH 8.0) with 1 mM GPP, 1 mM aromatic substrates, 5 mM MgCl_2_, and 0.9 g_cdw_ L^−1^ of cells for 24 h. The error range represents the standard deviation from biological replicates of n = 3. The absolute yield of NphB WT for **2a** was 6.4%, while the yields of NphB WT for **2b** and **2c** and F223Y* for **2d** were calculated based on the LC peak area due to the lack of available standards.

The designed variants were expressed in the *E. coli* and screened for the biotransformation of CBGA derivatives using **1a**∼**1d** and GPP as co-substrates (Figure 2). The NphB wild-type (WT) and NphB* were compared as references, and the identity of each substrate and corresponding product was confirmed by UHPLC-MS (Supplementary Figure S2). For product **2a**, corresponding to CBGA, a 68-fold and 2-fold improvement by NphB* S214T (S214T*) relative to the yield of WT and NphB*, respectively, were observed (Figure 2A). Other mutations mostly led to decreases in the yields of **2a**, including A232V* and F213Y*. A232I/L/M* and V271F* completely destroyed the biocatalytic activity. **2a** binding in the active site could have been interrupted by the placement of sterically oversized residues at this position.

**2b**, cannabigerovarin, was produced with a 5-fold decrease in yield by NphB* compared to WT (Figure 2B). A232I/L/M* and V271F* destroyed the activity observed for **2a**. Only S214T* could produce 1.8-fold and 8.6-fold improvements relative to WT and NphB*, respectively. The A232V*, T269I/L/V*, and V47A* yields were improved relative to NphB* but were still lower than WT.

Some mutations of **2c** improved yields more effectively than NphB*, namely, F213Y* and S214T*, with 1.9-fold and 1.5-fold increases, respectively. However, not only NphB* but also all the NphB*-based variants had limited yields compared to WT, with more than 3-fold decreases (Figure 2C). Most notably, **2d,** with no aliphatic branch, was not produced with either WT or NphB* but was produced by the NphB*-based variants. F213Y* was the most potent, followed by V271F* (Figure 2D). As an overall result of screening, S214T and F213Y were identified as key mutations that enhanced the activity of NphB* for the CBGA derivatives with various aliphatic chain lengths.

### 3.3 *In vitro* enzymatic conversion for the CBGA derivatives

**FIGURE 3:**
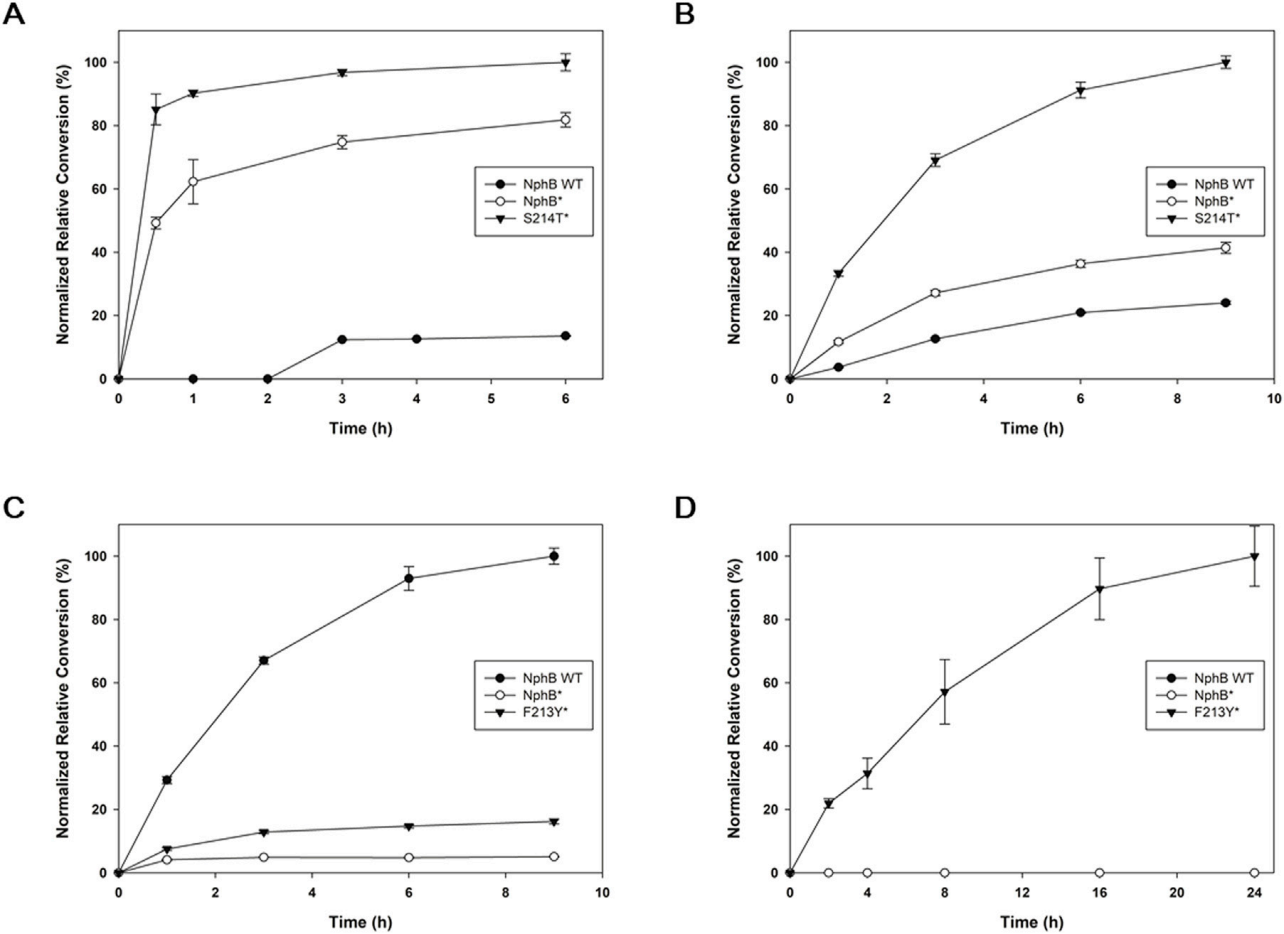
Time-dependent *in vitro* enzymatic conversion for the CBGA derivatives. NphB WT, NphB*, and the best-yielding NphB* variant for each of **2a**∼**2d** screened from the whole-cell biotransformation were purified and subjected to *in vitro* reaction by incubating 5 μM of purified enzyme with 5 mM MgCl_2_, 1.5 mM GPP, and 1 mM aromatic substrates at 30°C for 6–24 h. The highest detected yield for each product was set as 100%, and the results were normalized. **(A–D)**, the product **2a**∼**2d**, respectively. The error range represents the standard deviation from biological replicates of n = 3. The absolute yield of S214T* for **2a** corresponding to the 100% relative conversion was 47.0%, while 100% relative conversions for **2b**∼**2d** were calculated based on the LC peak area of the most productive variants due to the lack of available standards.

Because S214T* and F213Y* were found to be the yield-improved mutants for the CBGA derivatives, each of these variants, along with NphB WT and NphB*, was expressed and purified to confirm *in vitro* enzymatic activities on the derivatives (Figure 3; Supplementary Figure S3, S4). For **2a,** the tendency of the purified enzyme activity was similar to the whole-cell biotransformation, showing the highest conversion with S214T*, followed by NphB* and WT (Figure 3A). In the case of **2b**, the *in vitro* conversion was best in S214T*, followed by NphB* and WT, whereas the whole-cell biotransformation yield was in the order of S214T*, WT, and NphB* (Figure 3B). The activities for **2c** were in the order of WT, F213Y*, and NphB*, and the activity for **2d** was found only with F213Y*, a pattern similar to that found in the whole-cell biotransformation result (Figures 3C, D). The reactions for **2b**, **2c**, and **2d** were slower than **2a**, where the maximum conversions were reached in 9 h and 24 h windows for these derivatives compared to the 3 h window of **2a**.

Kinetic parameters such as *k*_*cat*_ and K_M_ were also investigated for NphB WT, NphB*, and S214T* against **2a**. The *k*_*cat*_ values for WT, NphB*, and S214T* were 0.020 ± 0.000 min^−1^, 0.944 ± 0.031 min^−1^, and 1.955 ± 0.402 min^−1^, respectively, and some correlation to catalytic distances (7.1 Å, 5.2 Å, and 4.8 Å, respectively, Supplementary Table S2) was found. However, not all variants followed the correlation, with shorter catalytic distances but lower activities. Although *k*_*cat*_ is influenced by catalytic distances, it is not solely determined by them. Other factors, such as enzyme conformational dynamics and the preorganized electrostatic environment of the active site, also play crucial roles in shaping catalytic efficiency by stabilizing transition states and facilitating substrate turnover (Warshel et al., 2006). The K_M_ values were 0.64 ± 0.03 mM, 0.17 ± 0.03 mM, and 0.49 ± 0.06 mM, respectively, which correlated to the docking scores of −6.12 kcal mol^−1^, −8.09 kcal mol^−1^, and −7.78 kcal mol^−1^ (Supplementary Table S2). The catalytic efficiency (*k*_*cat*_/K_M_) was 0.032, 5.821, and 3.940, which differed from the *in vitro* conversion results in Figure 3A, where S214T* exhibited the highest conversion. This discrepancy may be attributed to the higher *k*_*cat*_ of S214T* compared to NphB*, which provides an advantage at higher substrate concentrations by compensating for its higher K_M_.

### 3.4 Application of potent mutations on NphB WT

**FIGURE 4:**
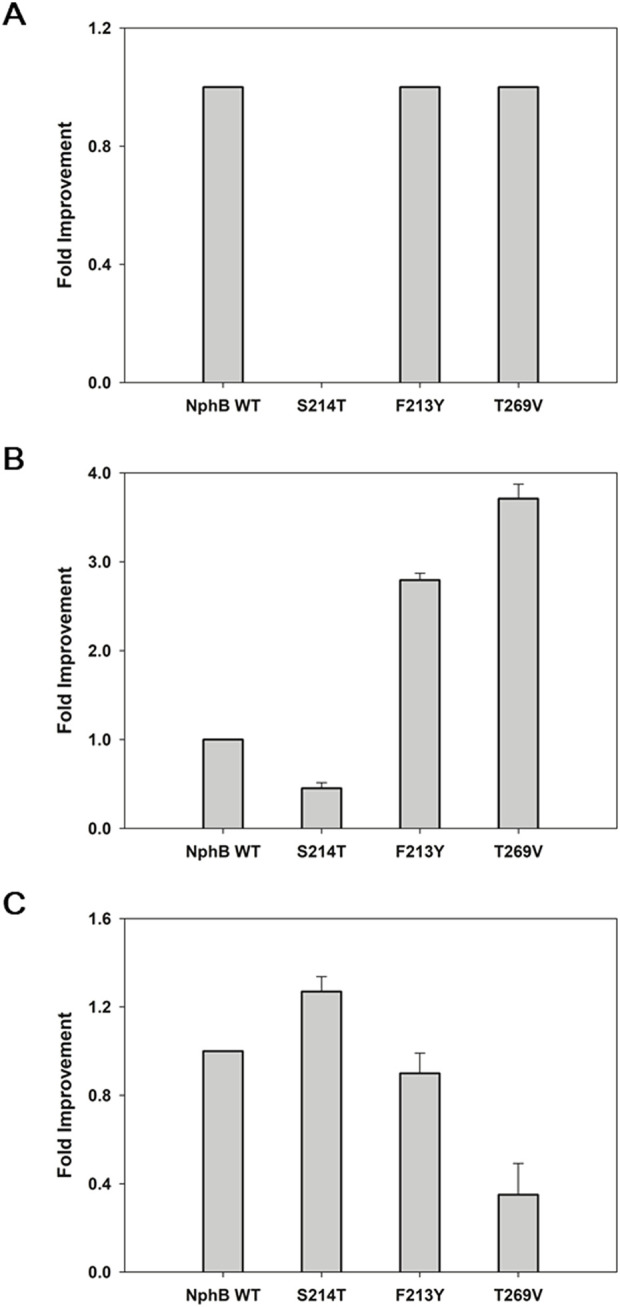
Biotransformation of the CBGA derivatives by NphB WT-based single mutants. **(A–C)**, fold improvement in the yields for **2a**∼**2c**, respectively, relative to the yield of NphB WT. No activity was found for **2d** in the NphB WT or WT-based single mutants investigated. The whole-cell biotransformation was performed at 30°C by incubating 0.9 g_cdw_ L^−1^ of cells with 5 mM MgCl_2_, 1 mM GPP, and 1 mM aromatic substrates in a 50 mM Tris-HCl buffer (pH 8.0) for 24 h. The error range represents the standard deviation from biological replicates of n = 3. The absolute yield of NphB WT for **2a** was 6.4%, while the yields of NphB WT for **2b** and **2c** were calculated based on LC peak area due to the lack of available standards.

As the yields of NphB* for **2b** and **2c** were lower than those of WT, it was postulated that some of the yield-enhancing mutants based on NphB* might lead to higher yields when applied to WT. S214T, F213Y, and T269V mutations were made accordingly, and the yield improvements were monitored for each product (Figure 4). F213Y and T269V did not show increased yields for **2a**, and the increase was negligible in S214T, demonstrating the G286S/Y288A mutations of NphB* are necessary for the efficient biotransformation of CBGA, and S214T* only works in conjunction with the double mutation (Figure 4A). The yields of **2b** were improved 3.7- and 2.8-fold in T269V and F213Y, whereas the yield was decreased by half in S214T (Figure 4B). This was unexpected as S214T* was the highest-yielding variant for **2b,** much higher than both WT and NphB*. This coincides with the dependency of S214T on the G286S/Y288A double mutation to enhance the activity. NphB S214T was the most active mutant for **2c,** with a 1.3-fold increase in the yield compared to WT and higher than all other NphB* variants (Figure 4C). As the length of the C6 aliphatic chain on the aromatic substrate decreased, S214T could increase the activity regardless of the NphB* double mutations. No activity was found for **2d** in WT or the WT-based single mutants as well as NphB*, implying the F213Y* or other NphB*-based additional mutations were critical for the biosynthesis of **2d**.

### 3.5 Structural analysis of the activity-enhanced NphB variants

**FIGURE 5:**
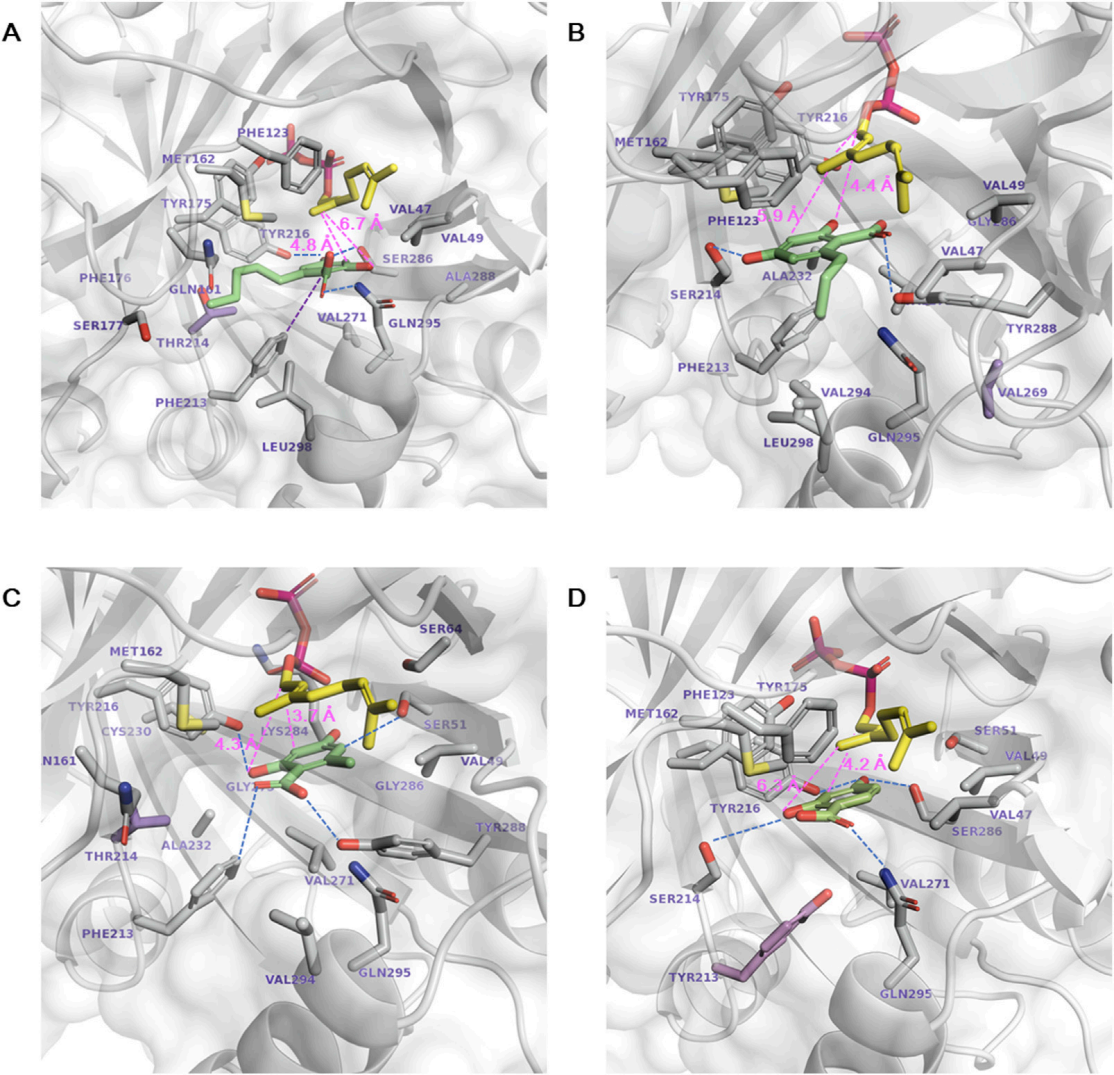
Docking of aromatic substrates in the active site of the highest-yielding variants. **(A) 1a** in S214T*, **(B) 1b** in T269V, **(C) 1c** in S214T, and **(D) 1d** in F213Y*. The stick representations are GPP (yellow), aromatic substrate (green), mutated residues (purple), and other substrate-interacting residues (gray). Hydrogen bonds and the catalytic distances between the C1 atom of GPP and the C3 or O2 atoms of the aromatic substrates are marked in dotted blue and pink lines, respectively.

The activity-enhanced S214T*, T269V, S214T, and F213Y* for each aromatic substrate **1a**, **1b**, **1c**, and 1d, respectively, were subjected to structural analysis by generating *in silico* models of these variants and docking the relevant substrates (Figure 5). **1a** was docked with decreased ΔG_bind_ in S214T* (−8.26 kcal mol^−1^) compared to NphB* (−8.09 kcal mol^−1^), even though the substrate binding conformations were similar, including the stabilization by F213, Y216, S286 and Q295 (Figures 1A, 5A). The catalytic distance between the GPP C1 atom and the **1a** C3 atom was also reduced from 5.2 Å to 4.8 Å. The mutated residue was able to enhance the hydrophobic interaction with the C6 aliphatic chain, indicated by the decreased minimum residue-ligand distance and the decreased free energy of van der Waals interaction contributed solely by the single mutated residue.

The **1b** binding pose in the T269V variant was distinctive as the direction of the C6 chain shifted from Y288 toward V294 and L298 due to the bulky tyrosine instead of the alanine as in NphB* (Figures 1B, 5B). The carboxylic acid moiety of **1b** instead faces toward Y288 and the O4 toward S214 for hydrogen bonding. Consequently, the O2 is in proximity to the C1 of GPP for the 2-O geranylated side product formation, explaining its higher yield than WT, NphB*, or even S214T*. Without the T269V mutation, NphB WT with **1b** resulted in the C3 being more proximal than O2 to the GPP C1 (Supplementary Figure S5). This also explains the high **1b** yield of WT over NphB* as the catalytic distance was reduced. More detailed effects of the T269V mutation on the **1b** binding in the active site require further dynamics studies because the residue position is distant from the substrate, and direct interaction was not observed due to masking by Y288.

**1c** in the NphB* and WT positions the C6 aliphatic chain on the opposite side, toward A288 in NphB* but away from Y288 in WT due to the bulky hydrophilic group (Figure 1C; Supplementary Figure S5). This results in catalytic distances of 5.0 Å and 3.8 Å in NphB* and WT, respectively, leading to the higher activity of WT. Furthermore, the WT-based single mutant S214T places **1c** deep inside the active site, with a reduced catalytic distance of 3.7 Å due to unfavorable repulsion between the weak aliphatic group of the T214 side chain and the O2 hydroxyl and carboxylic groups of **1c** (Figure 5C).

The activity for **1d** in F213Y* can be accounted for by the decreased catalytic distance and ΔG_bind_ (4.2 Å and −7.96 kcal mol^−1^) compared to NphB* (4.9 Å and −7.57 kcal mol^−1^) (Figures 1D, 5D). The larger tyrosine in place of phenylalanine could force **1d** toward GPP, sufficient to evoke activity for the substrate unreactive in the WT or NphB*. Overall, the activity-enhancing mutations were effective in terms of favorable repositioning of the aromatic substrates to be more proximal to GPP.

### 3.6 Optimization of the reaction condition

**FIGURE 6:**
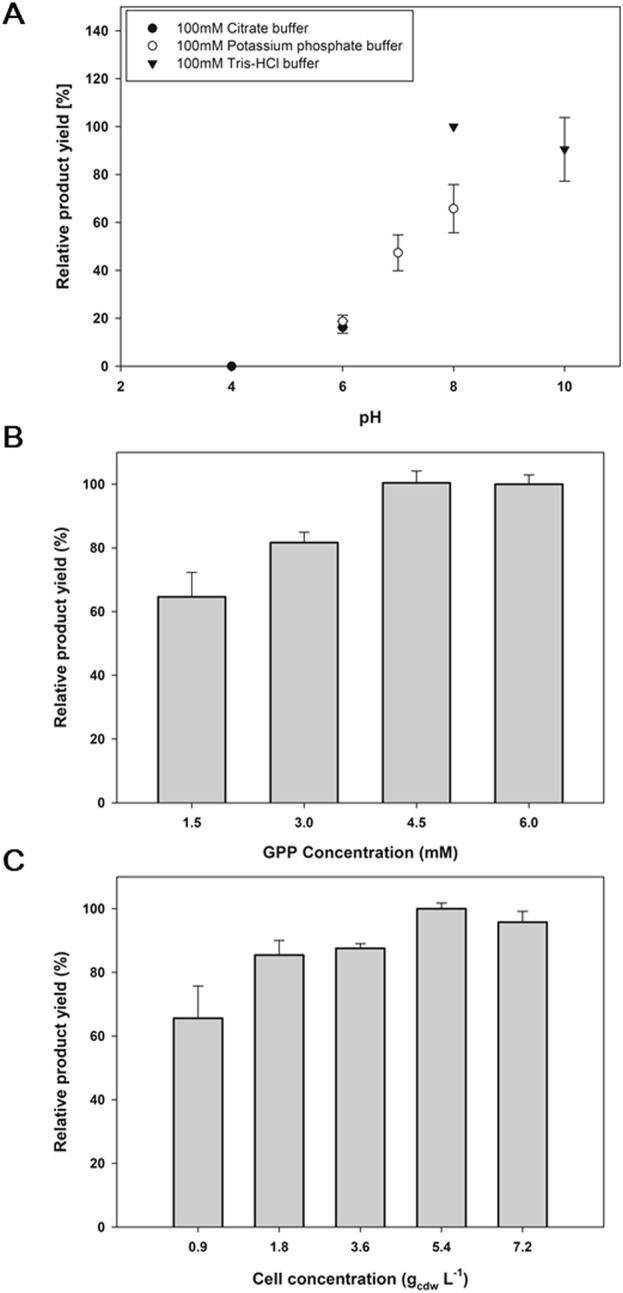
Optimization of the reaction condition for the biotransformation of **2a** by the S214T*-expressing whole-cell biocatalyst. **(A)** buffer PH, **(B)** concentration of GPP as a geranyl donor, and **(C)** biocatalyst concentration. Reaction A was carried out at 30°C with 1 mM GPP, 1 mM aromatic substrates, 5 mM MgCl_2_, and 0.9 g_cdw_ L^−1^ of cells for 24 h. Reaction B was performed using the optimized pH determined in **(A)**. Then, reaction **(C)** was conducted under the optimized pH and GPP concentration established in **(A)** and **(B)**. The error range represents the standard deviation from biological replicates of n = 3. 100% relative yields in **(A–C)** correspond to CBGA yields of 21.0%, 33.8%, and 45.6%, respectively.

**2a**, corresponding to CBGA, is a key platform compound in the biosynthesis of a wide range of cannabinoids. The reaction condition needs to be optimized for the efficient biotransformation of **2a**; hence, the reaction buffer pH, the concentration of GPP as a geranyl donor, and the whole-cell biocatalyst concentration were investigated. Among the pH range of 4.0∼10.0 that were tested, a pH 8.0 of 100 mM Tris—HCl buffer produced the highest product yield of 35% (Figure 6A). pH 6 and below decreased the yield more than 5-fold, while an alkaline pH 10 did not significantly affect the yield. GPP was applied in a range of 1.5∼6 mM concentrations along with 1 mM **1a**, where 4.5 mM and 6 mM of GPP led to a product yield of 34% (Figure 6B). The 4.5 mM GPP concentration was, therefore, thought to be optimal. For the whole-cell biocatalyst concentration, 5.4 g_cdw_ L^−1^ showed the highest product yield with 48% (Figure 6C). Consequently, the S214T*-expressing whole-cell biocatalyst of 5.4 g_cdw_ L^−1^ was reacted with 1 mM **1a** and 4.5 mM GPP in 100 mM Tris-HCl pH 8.0 for 6 h for a final yield of 48%. Under the same conditions, the conversion of **1b** with S214T* was 35%, and the conversions of **1c** and **1d** with F213Y* were 15% and 4%, respectively, which were the highest conversions achieved by the *E. coli* whole-cell biotransformation (Supplementary Figure S2; Qian et al., 2019).

## 4 Discussion

In this study, we successfully engineered NphB variants to enhance the biosynthesis of CBGA derivatives with diverse aliphatic chain lengths. By integrating computational modeling, site-directed mutagenesis, and biotransformation experiments, we elucidated key structure–function relationships of NphB and demonstrated its potential as a biocatalyst for cannabinoid production. The enzyme originally responsible for CBGA synthesis in *C. sativa* is geranyl-pyrophosphate-olivetolic acid geranyltransferase (GOT). There is no experimentally determined structure for GOT. Although UniProt provides an AlphaFold-predicted three-dimensional structure of GOT (ID: A0A455ZJC3), the structure includes a long N-terminal loop with very low confidence (pLDDT < 50), raising concerns about its reliability. A sequence-based homology search between GOT and *NphB* (ID: A0A2Z4JFA9) revealed no significant similarity. In fact, a BLAST search for sequences with more than 50% identity to *NphB* identified homologs exclusively from bacterial origins, including *Streptomyces*, *Actinacidiphila*, and *Mycobacterium*. The lack of reliable structural information and a high-throughput screening method for CBGA synthesis make both rational design and directed evolution strategies for GOT mutation engineering difficult. Additionally, the expression of plant enzymes in microbial hosts often results in low protein yields. As a result, GOT has not been characterized well, and most previous studies have focused on NphB instead of GOT for CBGA synthesis.

Our findings revealed that specific mutations, such as S214T and F213Y, play critical roles in improving yields and altering substrate specificity. S214T was particularly effective in reducing the catalytic distance and strengthening hydrophobic interactions with the C6 aliphatic chain, leading to enhanced production of CBGA (**2a**) and cannabigerovarin (**2b**). On the other hand, F213Y enabled the production of **2d**, a derivative with no aliphatic chain, by repositioning the substrate closer to GPP, thereby activating an otherwise unreactive substrate.

The results align with those of previous studies emphasizing the role of catalytic distance and substrate positioning in prenyltransferase activity (Qian et al., 2019; Valliere et al., 2019). These studies have focused on the regioselective production of CBGA over 2-O-geranlyated olivetolic acid and have successfully enhanced the activity of NphB for CBGA in the process. Our work expands this understanding by demonstrating how mutations can differentially affect substrates based on their aliphatic chain lengths. This insight provides a new framework for rational enzyme design targeting diverse cannabinoid derivatives.

Optimized reaction conditions further reinforced the practical potential of these engineered variants. By fine-tuning pH, GPP concentration, and biocatalyst density, we achieved a 48% yield of CBGA under optimal conditions, representing a significant improvement over previously reported biotransformation processes. This underscores the scalability of these engineered enzymes and their applicability to industrial cannabinoid production. However, certain limitations must be acknowledged. The dependency of S214T on the NphB* double mutation suggests that further studies are needed to decouple these interactions. Similarly, the reduced yield of **2c** in NphB* and F213Y* compared to WT highlights potential trade-offs introduced by certain mutations. Structural analysis indicated that these trade-offs likely stem from altered substrate positioning or pocket dynamics, which could be further explored using molecular dynamics simulations or high-throughput screening.

These results have significant implications for cannabinoid biosynthesis. Plant-derived enzymes face challenges such as low scalability and limited substrate versatility, whereas microbial expression systems, coupled with engineered enzymes, offer a more sustainable and efficient alternative (Andre et al., 2016; Lim et al., 2022). Our findings demonstrate that rationally designed NphB variants can expand the cannabinoid repertoire, providing a platform for producing novel derivatives with therapeutic potential. For example, the production of **2d** by F213Y* opens possibilities for generating cannabinoids with unique properties that were previously inaccessible through wild-type enzymes.

Several avenues warrant further exploration. Engineering NphB for broader substrate tolerance could lead to the biosynthesis of entirely novel cannabinoids. Additionally, combining these engineered enzymes with microbial chassis optimized for precursor production, such as glucose-derived isoprenoids and aromatic substrates, could enable one-pot biosynthesis, streamlining the production process (Tan et al., 2018; Ward et al., 2018; Han et al., 2023). The development of high-throughput screening methods for combinatorial mutations may also uncover synergistic effects that further enhance catalytic efficiency. Finally, future research should investigate the structural basis of observed activity enhancements, particularly the role of residue-specific interactions in substrate positioning.

In conclusion, this study establishes a foundation for expanding the cannabinoid repertoire through enzyme engineering. By identifying key mutations and optimizing reaction conditions, we demonstrated the potential of NphB variants to efficiently produce CBGA derivatives. These findings not only advance our understanding of aromatic prenyltransferases but also provide a roadmap for developing biocatalysts tailored for industrial cannabinoid production. With further refinement, these engineered enzymes could significantly impact the sustainable and scalable production of cannabinoids with diverse therapeutic applications.

## Data Availability

The original contributions presented in the study are included in the article/Supplementary Material, further inquiries can be directed to the corresponding authors.
