# Supplementary material for: Biosynthesis of novel cannabigerolic acid derivatives by engineering the substrate specificity of aromatic prenyltransferase
Source: Front Bioeng Biotechnol. 2025 Apr 10;13:1563708. doi: 10.3389/fbioe.2025.1563708 (PMC12018323; doi:10.3389/fbioe.2025.1563708)
Supplement: Supplementary file 1 [file DataSheet1.docx]

Supplementary Material


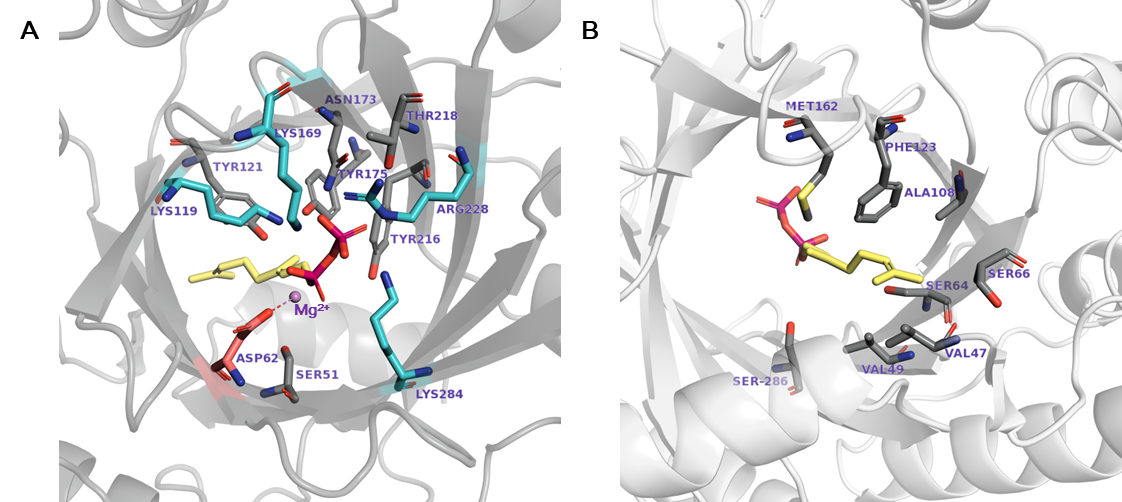


**Supplementary Figure 1.** Docking of GPP in the NphB* active site showing: (A) diphosphate moiety-interacting residues; (B) geranyl moiety-interacting residues. The positively charged residues forming a salt bridge network with the diphosphate moiety and the negatively charged Asp62 coordinating the Mg^2+^ ion are colored as skyblue and pink, respectively.

**A**


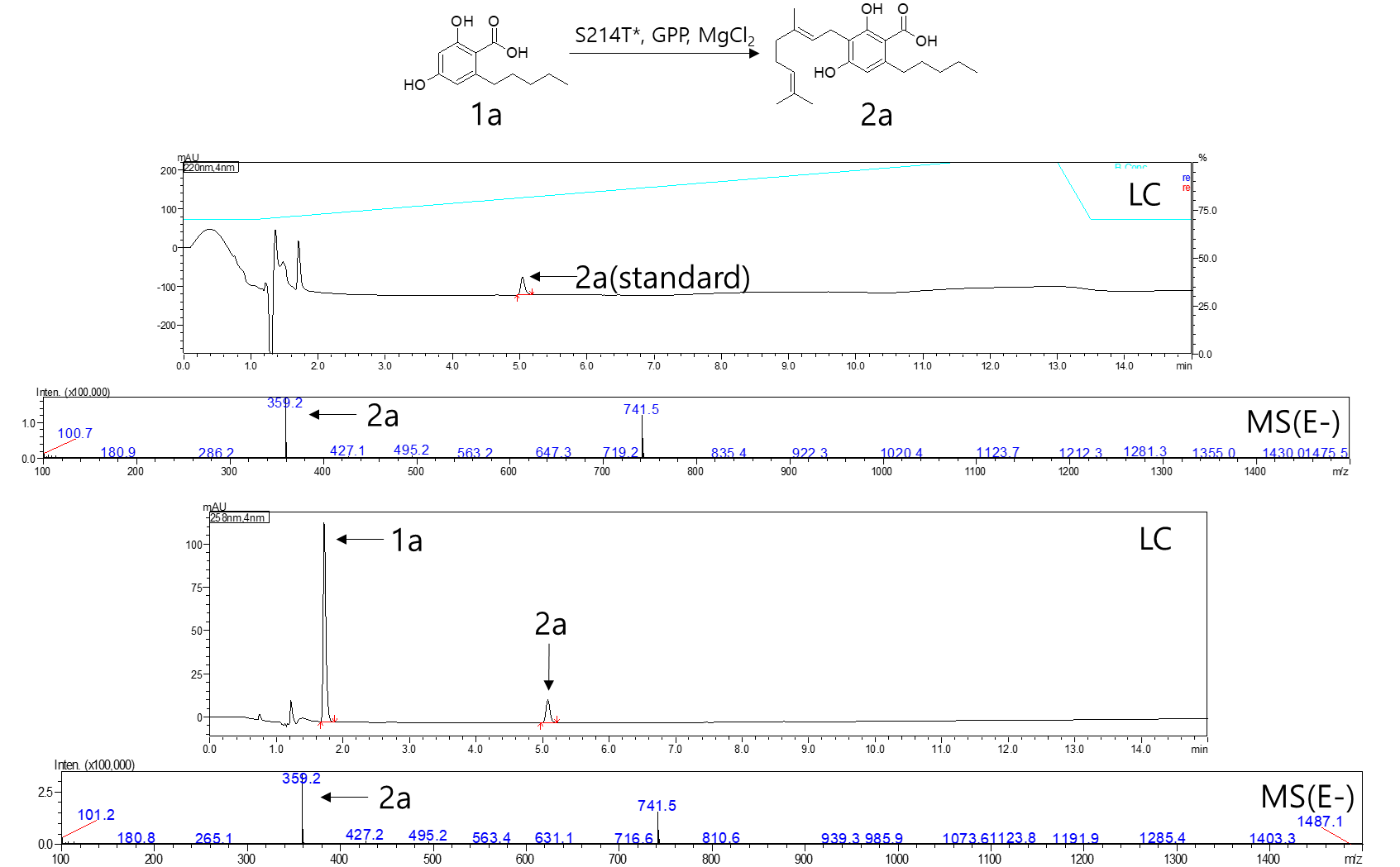


**B**

**
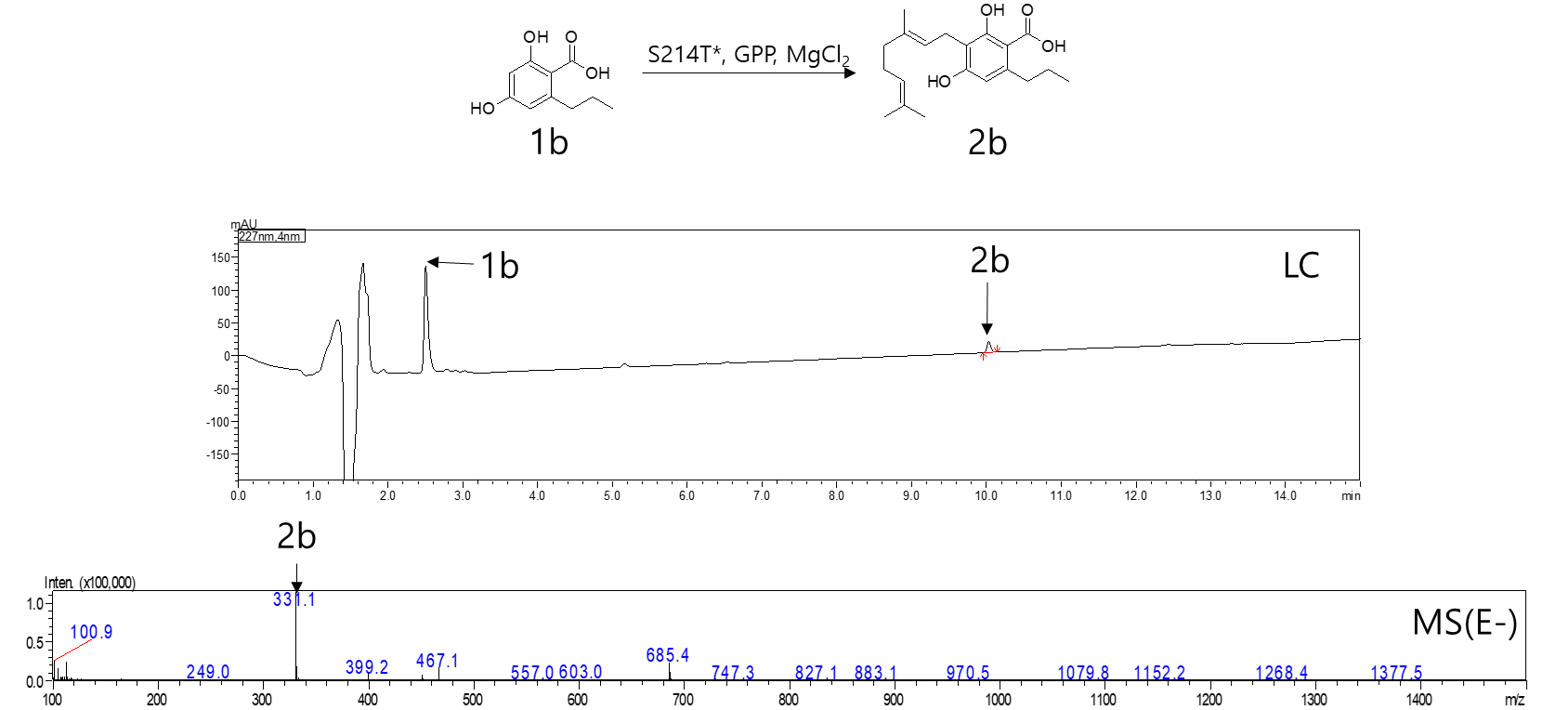
**

**C**


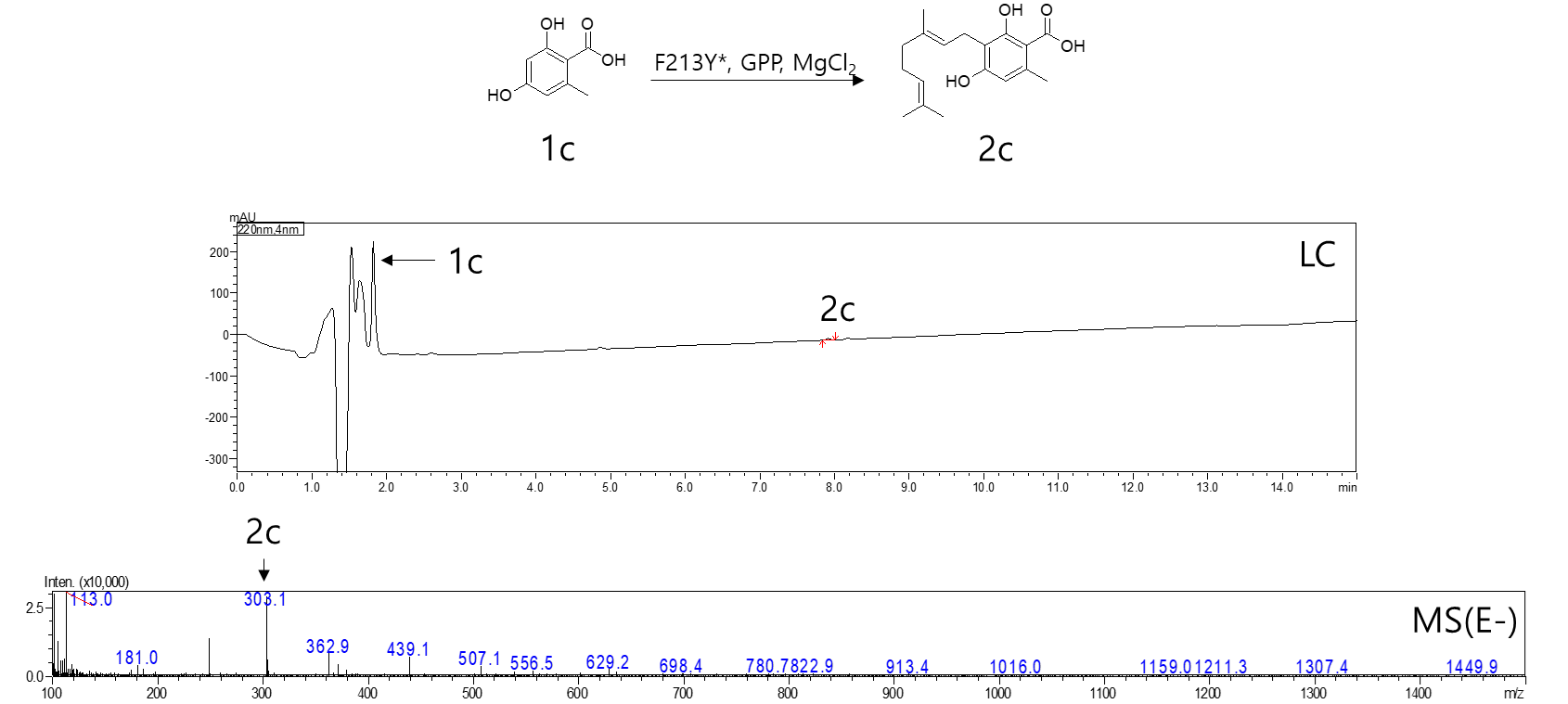


**D**


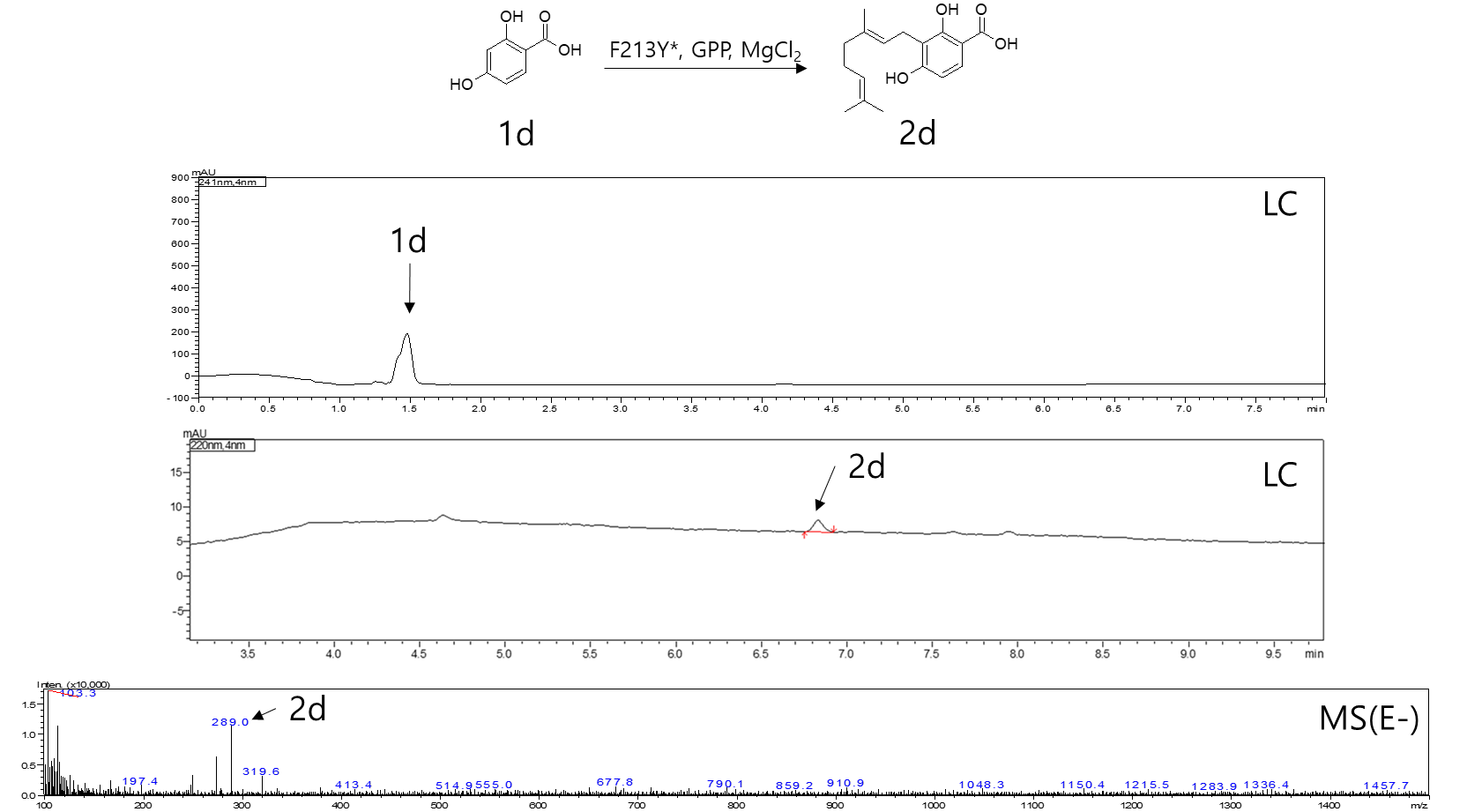


**Supplementary Figure 2.** Conversion of the aromatic substrates **1a**~**1d** to the CBGA derivatives **2a~2d** in the scheme 1, confirmed by UHPLC-mass spectrometry (panel A~D, respectively). The chromatogram and mass spectrum for a CBGA standard is shown in the panel A for comparison.

**A**


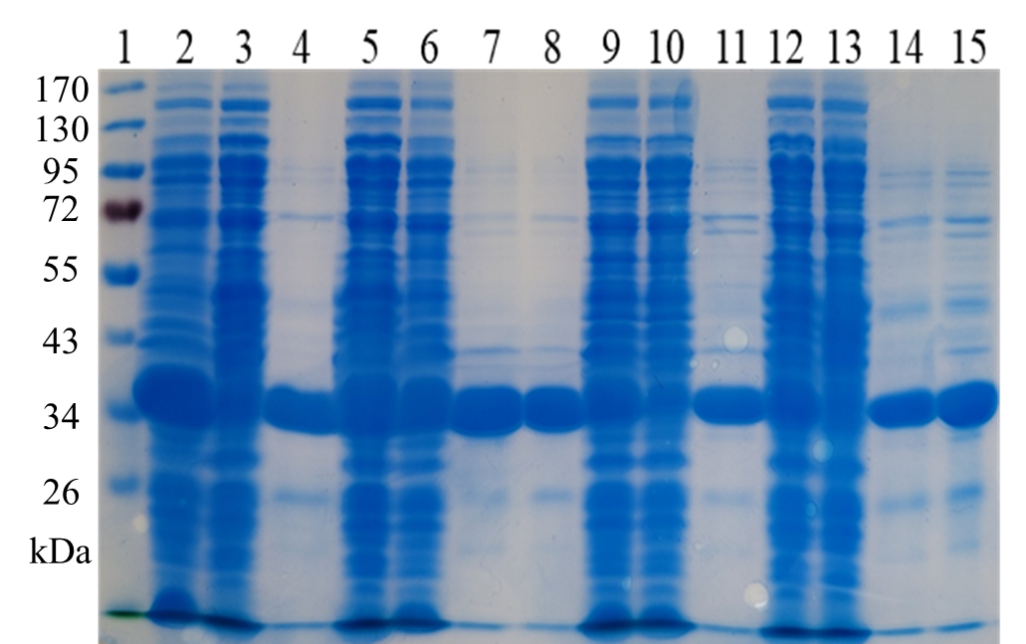


**B**


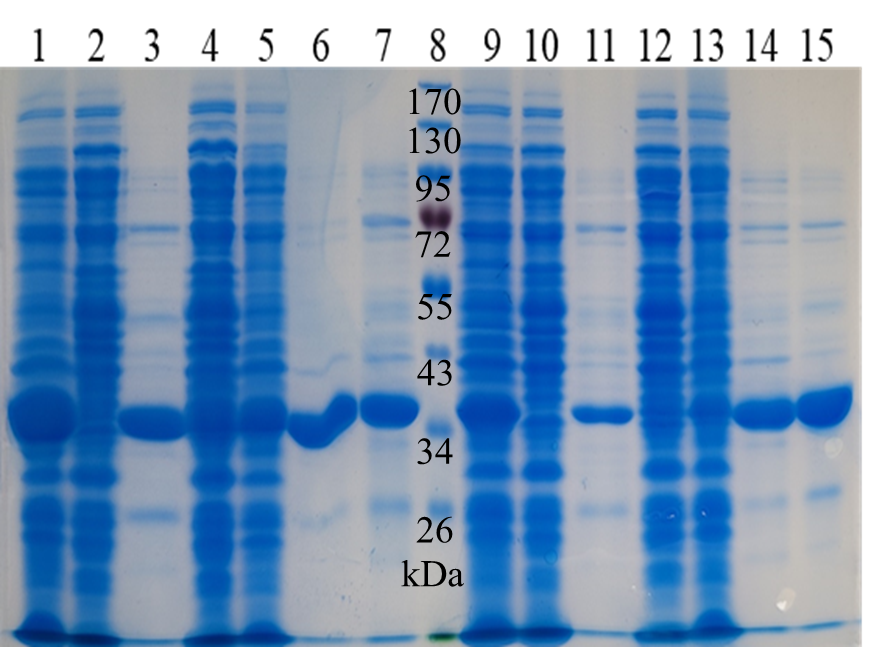


**Supplementary Figure 3.** Expression and purification of NphB variants. (A) Lane 1: ladder. Lane 2,5: soluble fraction of BL21(DE3) expressing NphB. Lane 3, 6: unpurified NphB sample. Lane 4, 7: purified NphB sample. Lane 8: collected purified NphB sample. Lane 9, 12: soluble fraction of BL21(DE3) expressing NphB*. Lane 10, 13: unpurified NphB* sample. Lane 11, 14: purified NphB* sample. Lane 15: collected purified NphB* sample. (B) Lane 1, 4: soluble fraction of BL21(DE3) expressing S214T*. Lane 2, 5: unpurified S214T* sample. Lane 3, 6: purified S214T* sample. Lane 7: collected purified S214T* sample. Lane 8: ladder. Lane 9, 12: soluble fraction of BL21(DE3) expressing F213Y*. Lane 10, 13: unpurified F213Y* sample. Lane 11, 14: purified F213Y* sample. Lane 15: collected purified F213Y* sample.


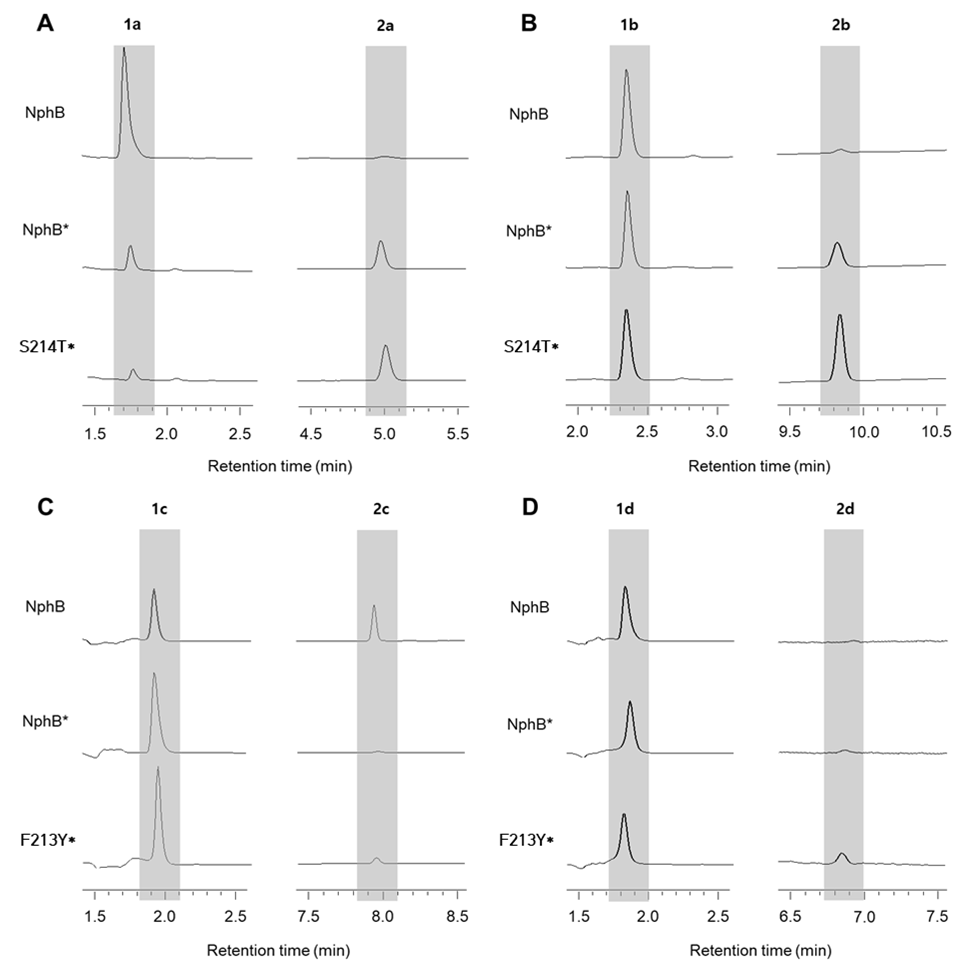


**Supplementary Figure 4.** UHPLC peaks for the *in vitro* enzymatic conversion of the CBGA derivatives. A~D, 9h conversion of the aromatic substrates **1a**~**1d** to the geranylated, CBGA derivative products **2a**~**2d** by the purified NphB WT, NphB* and the best performing NphB* variants, respectively.


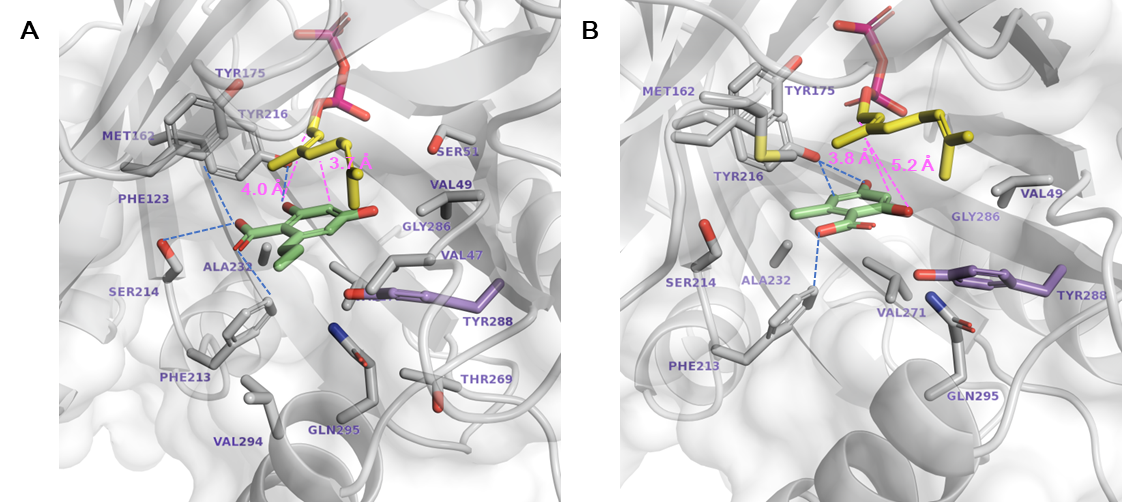


**Supplementary Figure 5.** Docking of **1b** and **1c** in the active site of NphB WT. The two substrates showed higher activity in NphB WT than NphB*. A, **1b**; B, **1c.** The stick representations are: GPP (yellow); aromatic substrate (green); G286/Y288 (purple); other substrate-interacting residues (gray). Hydrogen bonds and the catalytic distances (between the C1 atom of GPP, and the C3 or the O2 atom of aromatic substrate) are marked in blue- and pink-dotted lines, respectively.

**Supplementary Table 1.** Primers used in this study

| **Primer** | **Sequence (5’→ 3’)** |
| --- | --- |
| Cloning |  |
| NphB Ins For | TGC ATC ATC ATC ATC ATC ACA GCA GCG GCA TGA GCG AAG CAG CTG ATG T |
| NphB Ins Rev | ACG GAG CTC GAA TTC TCA GTC TTC AAG GCT GTC GAA GGC |
| Vector For | GAA TTC GAG CTC CGT CGA CAA GCT T |
| Vector Rev | GTG ATG ATG ATG ATG ATG CAT ATG TAT ATC TCC |
| Mutation |  |
| NphB* For | CCG AAA GAG GAG TAT TAC AAA TTA tct GCT gca TAT CAT ATA ACA |
| NphB* Rev | ATC TGT TAT ATG ATA tgc AGC aga TAA TTT GTA ATA CTC CTC T |
| V47L For | GGA TCT ctt GTG GTA TTC TCG ATG GCT |
| V47L Rev | TAC CAC aag AGA TCC CCC CTC TAC TAA |
| V47M For | GGA TCT atg GTG GTA TTC TCG ATG GCT |
| V47M Rev | TAC CAC cat AGA TCC CCC CTC TAC TAA |
| V47I For | GGA TCT att GTG GTA TTC TCG ATG GCT |
| V47I Rev | TAC CAC aat AGA TCC CCC CTC TAC TAA |
| V47A For | GGA TCT gct GTG GTA TTC TCG ATG GCT |
| V47A Rev | TAC CAC agc AGA TCC CCC CTC TAC TAA |
| V47K For | GGA TCT aaa GTG GTA TTC TCG ATG GCT |
| V47K Rev | TAC CAC ttt AGA TCC CCC CTC TAC TAA |
| V47R For | GGA TCT cgt GTG GTA TTC TCG ATG GCT |
| V47R Rev | TAC CAC acg AGA TCC CCC CTC TAC TAA |
| V49L For | GTT GTG cta TTC TCG ATG GCT AGT GGT |
| V49L Rev | CGA GAA tag CAC AAC AGA TCC CCC CTC |
| V49M For | GTT GTG atg TTC TCG ATG GCT AGT GGT |
| V49M Rev | CGA GAA cat CAC AAC AGA TCC CCC CTC |
| V49I For | GTT GTG ata TTC TCG ATG GCT AGT GGT |
| V49I Rev | CGA GAA tat CAC AAC AGA TCC CCC CTC |
| V49A For | GTT GTG gca TTC TCG ATG GCT AGT GGT |
| V49A Rev | CGA GAA tgc CAC AAC AGA TCC CCC CTC |
| V49K For | GTT GTG aaa TTC TCG ATG GCT AGT GGT |
| V49K Rev | CGA GAA ttt CAC AAC AGA TCC CCC CTC |
| V49R For | GTT GTG cga TTC TCG ATG GCT AGT GGT |
| V49R Rev | CGA GAA tcg CAC AAC AGA TCC CCC CTC |
| F213Y For | CGT TCA tac AGT GTA TAT CCT ACT CTT |
| F213Y Rev | TAC ACT gta TGA ACG CTT ACA AAA TTT |
| S214T For | TCA TTC act GTA TAT CCT ACT CTT AAC |
| S214T Rev | ATA TAC agt GAA TGA ACG CTT ACA AAA |
| S214A For | TCA TTC gct GTA TAT CCT ACT CTT AAC |
| S214A Rev | ATA TAC agc GAA TGA ACG CTT ACA AAA |
| Y216F For | AGT GTA ttt CCT ACT CTT AAC TGG GAA |
| Y216F Rev | AGT AGG aaa TAC ACT GAA TGA ACG CTT |
| Y216M For | AGT GTA atg CCT ACT CTT AAC TGG GAA |
| Y216M Rev | AGT AGG cat TAC ACT GAA TGA ACG CTT |
| Y216L For | AGT GTA ctt CCT ACT CTT AAC TGG GAA |
| Y216L Rev | AGT AGG aag TAC ACT GAA TGA ACG CTT |
| Y216I For | AGT GTA att CCT ACT CTT AAC TGG GAA |
| Y216I Rev | AGT AGG aat TAC ACT GAA TGA ACG CTT |
| A232I For | TGC TTT ata GTT ATC TCA AAT GAT CCA |
| A232I Rev | GAT AAC tat AAA GCA AAG CCG GTC GAT |
| A232L For | TGC TTT tta GTT ATC TCA AAT GAT CCA |
| A232L Rev | GAT AAC taa AAA GCA AAG CCG GTC GAT |
| A232M For | TGC TTT atg GTT ATC TCA AAT GAT CCA |
| A232M Rev | GAT AAC cat AAA GCA AAG CCG GTC GAT |
| A232V For | TGC TTT gta GTT ATC TCA AAT GAT CCA |
| A232V Rev | GAT AAC tac AAA GCA AAG CCG GTC GAT |
| T269M For | AAA CGC atg CTT GTT TAC GGT TTG ACC |
| T269M Rev | AAC AAG cat GCG TTT CTC TCC GAC ATA |
| T269I For | AAA CGC atc CTT GTT TAC GGT TTG ACC |
| T269I Rev | AAC AAG gat GCG TTT CTC TCC GAC ATA |
| T269L For | AAA CGC ctc CTT GTT TAC GGT TTG ACC |
| T269L Rev | AAC AAG gag GCG TTT CTC TCC GAC ATA |
| T269V For | AAA CGC gtc CTT GTT TAC GGT TTG ACC |
| T269V Rev | AAC AAG gac GCG TTT CTC TCC GAC ATA |
| V271F For | ACC CTT ttt TAC GGT TTG ACC TTA TCC |
| V271F Rev | ACC GTA aaa AAG GGT GCG TTT CTC TCC |

*The parts where the vector and insert overlap are indicated with underlines. Codons for designated mutations are indicated in lowercase letters. For and Rev refer to forward and reverse primers, respectively.

**Supplementary Table 2.** Docking scores and catalytic distances for NphB variants and CBGA derivatives.

| CBGA Derivatives | Variant | Docking Score  (kcal mol^-1^) | Catalytic Distance^1^  (Å) |
| --- | --- | --- | --- |
| **1a** | NphB WT | -6.12 | 7.1 |
|  | NphB* | -8.09 | 5.2 |
|  | A232I* | N.P.^2^ | N.P. |
|  | A232L* | N.P. | N.P. |
|  | A232M* | N.P. | N.P. |
|  | A232V* | -7.72 | 5.0 |
|  | F213Y* | -7.68 | 5.1 |
|  | S214T* | -7.78 | 4.8 |
|  | V271F* | N.P. | N.P. |
|  | S214T | N.P. | N.P. |
|  | F213Y | -6.46 | 5.6 |
|  | T269V | N.P. | N.P. |
| **1b** | NphB WT | -6.61 | 4.4 |
|  | NphB* | -5.00 | 4.9 |
|  | A232I* | N.P. | N.P. |
|  | A232L* | N.P. | N.P. |
|  | A232M* | N.P. | N.P. |
|  | A232V* | -5.72 | 4.9 |
|  | F213Y* | N.P. | N.P. |
|  | S214T* | -7.74 | 5.2 |
|  | T269I* | -5.88 | 5.7 |
|  | T269L* | -5.98 | 5.1 |
|  | T269V* | -6.61 | 5.8 |
|  | V271F* | N.P. | N.P. |
|  | V47A* | -5.72 | 5.8 |
|  | S214T | -6.10 | 5.7 |
|  | F213Y | -7.48 | 5.0 |
|  | T269V | -8.14 | 5.9 |
| **1c** | NphB WT | -6.70 | 3.8 |
|  | NphB* | -5.90 | 5.0 |
|  | A232I* | N.P. | N.P. |
|  | A232L* | N.P. | N.P. |
|  | A232M* | N.P. | N.P. |
|  | A232V* | N.P. | N.P. |
|  | F213Y* | -6.30 | 5.2 |
|  | S214T* | -6.01 | 5.6 |
|  | V271F* | N.P. | N.P. |
|  | V47I* | -5.97 | 5.4 |
|  | V47L* | -5.95 | 6.7 |
|  | V47M* | -5.87 | 6.1 |
|  | V49I* | -6.07 | 6.5 |
|  | V49L* | -5.94 | 5.7 |
|  | V49M* | -5.75 | 5.3 |
|  | S214T | -6.76 | 3.7 |
|  | F213Y | -6.91 | 5.2 |
|  | T269V | -6.25 | 5.4 |
| **1d** | NphB WT | N.P. | N.P. |
|  | NphB* | -4.24 | 4.9 |
|  | A232I* | -6.43 | 4.5 |
|  | A232L* | -6.49 | 5.1 |
|  | A232M* | -6.41 | 6.5 |
|  | A232V* | -6.25 | 4.2 |
|  | F213Y* | -7.95 | 4.2 |
|  | S214T* | -5.80 | 5.7 |
|  | T269I* | N.P. | N.P. |
|  | T269L* | N.P. | N.P. |
|  | T269M* | -5.68 | 4.6 |
|  | V271F* | -6.82 | 4.8 |
|  | V47I* | -4.34 | 7.1 |
|  | V47K* | N.P. | N.P. |
|  | V47L* | -6.39 | 5.7 |
|  | V47M* | -6.48 | 5.8 |

^1^Catalytic distance refers to the distance between C1 atom of GPP and C3 atom of aromatic substrate where a covalent bond is formed during the ligation reaction.

^2^N.P. refers to no productive binding pose produced from docking
